# Cross-organ sensitization between the prostate and bladder in an experimental rat model of lipopolysaccharide (LPS)-induced chronic pelvic pain syndrome

**DOI:** 10.1186/s12894-021-00882-9

**Published:** 2021-08-21

**Authors:** Ozgu Aydogdu, Pinar Uyar Gocun, Patrik Aronsson, Thomas Carlsson, Michael Winder

**Affiliations:** 1 Department of Pharmacology, Institute of Neuroscience and Physiology, The Sahlgrenska Academy, University of Gothenburg, Gothenburg, Sweden; 2 Department of Pathology, School of Medicine, Gazi University, Ankara, Turkey

**Keywords:** Lipopolysaccharide (LPS), CPPS, Prostate, Bladder, LUTS, Overactive bladder

## Abstract

**Background:**

The aim of the current study was to investigate the effects of chronic prostatitis/chronic pelvic pain syndrome (CP/CPPS) on bladder function via prostate-to-bladder cross-sensitization in a rat model of lipopolysaccharide (LPS)-induced prostate inflammation.

**Methods:**

Male rats were intraprostatically injected with LPS or saline, serving as control. Micturition parameters were examined in a metabolic cage 10 or 14 days later. Subsequently, to evaluate bladder function, cystometry was performed. Micturition cycles were induced by saline infusion and cholinergic and purinergic contractile responses were measured by intravenous injection with methacholine and ATP, respectively. Thereafter, the prostate and bladder were excised and assessed histopathologically for possible inflammatory changes.

**Results:**

Metabolic cage experiments showed increased urinary frequency in rats with LPS-induced CP/CPPS. Cystometry showed a significant increase in the number of non-voiding contractions, longer voiding time and lower compliance in CP/CPPS animals compared to controls. Induction of CP/CPPS led to significantly reduced cholinergic and purinergic bladder contractile responses. Histopathological analysis demonstrated prostatic inflammation in CP/CPPS animals. There were no significant differences between the groups regarding the extent or the grade of bladder inflammation. Prostate weight was not significantly different between the groups.

**Conclusions:**

The present study shows that prostate-to-bladder cross-sensitization can be triggered by an infectious focus in the prostate, giving rise to bladder overactivity and alterations in both afferent and efferent signalling. Future studies are required to fully understand the underlying mechanisms.

## Introduction

Chronic prostatitis/chronic pelvic pain syndrome (CP/CPPS) constitutes an important part of urology visits [1]. Despite rare association between bacterial foci in the prostate and CP/CPPS, infection has for long been speculated to be a potential factor that can trigger inflammatory pathways in the pelvic region [1, 2]. Some studies have demonstrated the presence of bacterial DNA in prostate tissues from men with CP/CPPS [1, 3, 4] and it has been suggested that an initial intraprostatic bacterial colonization could provoke chronic inflammation that possibly entails insistent structural changes in the prostate [5].

A large number of male patients diagnosed with CP/CPPS suffer from lower urinary tract symptoms (LUTS), including urgency and frequency, similar to patients with overactive bladder (OAB) [6, 7]. The potential mechanisms by which chronic prostatic inflammation contributes to LUTS has recently been highlighted [6, 8–10]. Cross-organ sensitization between the urinary bladder and prostate has been speculated as a potential mechanism to explain the possible negative effects of chronic prostate inflammation on bladder function [10–12]. A recent study on zymosan-induced CP/CPPS in rats supports this notion [13].

Interstitial cystitis/bladder pain syndrome (IC/BPS) is often referred to as a disease that more commonly affects women [14]. Nevertheless, studies have suggested that the true prevalence of IC/BPS in men is higher than thought, which may be due to an underdiagnosis of this pathology in male patients [15, 16]. I.e., that many men diagnosed with CP/CPPS may also have concomitant IC/BPS. It is noteworthy that male patients suffering from CP/CPPS frequently experience inadequate treatment [17]. Besides a lack of effective medication, possible negative effects of chronic prostate inflammation on urinary bladder function and urothelial pathophysiology may explain the unsatisfactory management of men with CP/CPPS. Considering the relatively large number of patients who do not respond positively to treatment for CP/CPPS, more effort is required to find an explanation for the potential cross-organ sensitization between prostate and bladder. For this, it is necessary to understand possible underlying mechanisms for inflammatory processes in the pelvic region. This will potentially lead to the discovery of new and more effective pharmacological targets for the treatment of male patients with CP/CPPS.

In the present study, it was hypothesized that an intraprostatic infectious focus could be an inducing factor for CP/CPPS which subsequently leads to pelvic cross-sensitization. For this, prostatic inflammation was induced in male rats by intraprostatic injection with lipopolysaccharide (LPS), a component of the outer layer of the cell wall of gram-negative bacteria. LPS can activate the immune system and cause chronic inflammation, which in turn can cause release of various pro-inflammatory cytokines including interleukin-1 (IL-1), interleukin-6 (IL-6) and tumor necrosis factor alpha (TNF-α) [2]. Micturition parameters were studied in a metabolic cage at 10 and 14 days after LPS injection. Subsequently, urodynamic properties were examined during cystometry by repeated bladder filling. Further, methacholine (MeCh) and adenosine-5′-triphosphate (ATP) were used to examine cholinergic and purinergic responses, respectively. Lastly, inflammatory changes in the prostate and urinary bladder were studied immunohistochemically.

## Materials and methods

The current study aimed to investigate if bacterially induced chronic prostate inflammation leads to symptoms of bladder overactivity. Further, the study aimed to examine the potential underlying mechanisms causing these alterations. Eighteen adult male Sprague–Dawley rats (300–450 g; Charles River Laboratories, Calco, Italy) were used in the current study which was approved by the local ethics committee at the University of Gothenburg, Sweden (permit number: 1794/2018). All experiments were designed to minimize the suffering of the animals during and after the surgical and experimental procedures. The number of animals were chosen based on previous studies and to ensure obtainment of objective and reliable results, i.e. avoid underpowering, but at the same time minimize the number of animals used, i.e. avoid overpowering. All drugs were purchased from Sigma-Aldrich, St Louis, USA unless otherwise stated.

### Study design

**Fig. 1:**
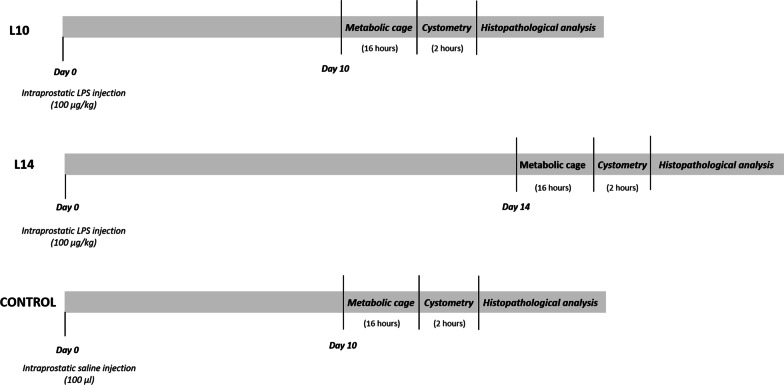
Study flowcharts. Timelines for the experimental procedures in each treatment group. Male rats were injected intraprostatically with either lipopolysaccharide (LPS; (L10 and L14; 100 µg * kg^−1^) or saline (Control; 100 µL). After 10 (L10, Control) or 14 (L14) days the rats were placed in a metabolic cage for 16 h. Subsequently, cystometry was performed. This was followed by histopathological analysis of bladder and prostate tissues

The animals were randomly divided into three groups (n = 6 in each group; Fig. 1). In group 1 and 2, LPS (100 µL; 100 µg * kg^−1^) was injected into the dorsal and ventral lobes of the prostate (50 µL in each lobe). In group 3, the rats were similarly injected with vehicle (100 µL saline, serving as control). During intraprostatic injections, the needle tip was tunnelled 2–3 mm subcapsularly and the needle was held in injection site for approximately 20 s as a standard procedure to avoid a possible leakage from the prostate. All intraprostatic injections were performed with laparotomy under deep anaesthesia with 3% isoflurane on a thermo-regulated heating pad. Following the injection procedure, abdominal muscle and skin were closed with separate surgical sutures. A single dose of subcutaneous buprenorphine (0.1 mg * kg^−1^) was used as postoperative analgesia.

### Metabolic cage

Ten or 14 days after intraprostatic LPS injection (termed as group L10 and L14, respectively) or 10 days after intraprostatic saline injection (control), metabolic cage experiments were carried out. The time intervals were chosen based on previous observations of inflammatory responses in the prostate [2, 13]. These previous studies demonstrate possible differences at 10 and 14 days after intraprostatic LPS injection, respectively. Further, they indicate that 7 days may be too short of an interval for observations of prostatitis after a single injection and that differences that are seen after 14 days are not significantly increased at later time points (i.e. after 21 days). Similar to previous studies, the animals were currently placed in a metabolic cage with free access to water and the voided urine was collected continuously for 16 h, typically from 4 PM to 8 AM. [18, 19]. The total water intake and total amount of expelled urine were measured. For registration of the number of micturitions, a laser Doppler (WFL30-40B416; SICK, Richmond Hill, Canada) that registered each drop of voided urine was used. This allowed for micturition frequency and voided volume per micturition to be calculated. A MP150WSW data acquisition system and the AcqKnowledge 3.8.1 software (BioPac Systems, Goleta, USA) were used to record the metabolic cage data.

### Cystometry

Immediately following each metabolic cage experiment, cystometry was performed. Deep anaesthesia was induced with 3% isoflurane and maintained during the entirety of the cystometry investigations. The surgical procedure followed previous similar studies [20]. Briefly, the urinary bladder was exposed by laparotomy and the femoral artery and vein were catheterized to monitor blood pressure and administer drugs (MeCh and ATP), respectively. After the catheterization of the femoral blood vessels, a pressure sensing catheter and a cannula were placed in the urinary bladder via a midline incision in the bladder dome and subsequently fixed with a ligature. Saline was infused via the cannula to induce simulated micturition cycles. The bladder was emptied before each infusion and was filled until the rat voided (approx. 30–40 s). The change in intravesical pressure that occurred during bladder filling (ΔP), volume change in the bladder (ΔV), voiding time and non-voiding contractions (NVCs), defined as increases in intravesical pressure more than 10 mmHg over baseline pressure without any voiding, were noted during the experiments. For standardization purposes, NVCs were counted during a two hour period, from the beginning of the first induced micturition cycle and onwards. Bladder compliance was calculated by dividing ΔV by ΔP. Simulated micturition cycles were performed five times before and after concentration–response series of the cholinergic agonist MeCh (1, 2 and 5 µg * kg^−1^ i.v.) and the purinergic agonist ATP (5, 10 and 100 µg * kg^−1^ i.v.). Each concentration–response series was performed two times. Calculations were performed on the average responses during the induced micturition cycles and the concentration–response series.

### Histopathology

**Fig. 2:**
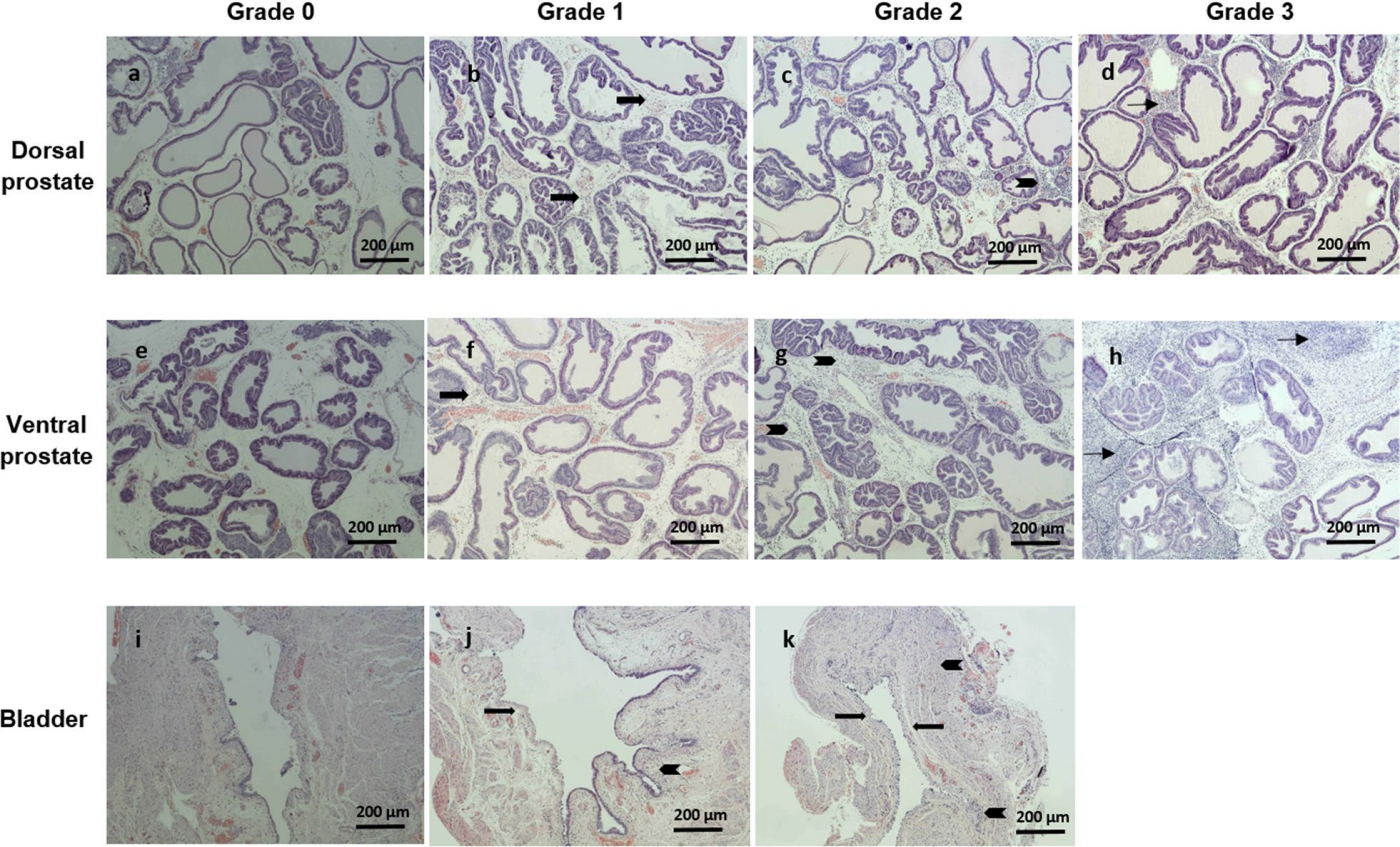
Examples of inflammation scoring (grade). All tissues were stained with haematoxylin–eosin and examined at 10X (a-j) or 4X (k) magnification. **a** No signs of inflammatory cells. **b** Individual inflammatory cells (arrows) separated by distinct interventing spaces are seen around the glands (< 100 cells/mm^2^). **c** Confluent sheets of inflammatory cells (arrowhead) around the glands without tissue destruction or lymphoid nodule/follicle formation (100–500 cells/mm^2^). **d** Confluent sheets of inflammatory cells with lymphoid nodule formation (arrow) lie within stroma around the glands (> 500 cells/mm^2^). **e** No signs of inflammatory cells. **f** Few inflammatory cells (arrow) seen around the glands (< 100 cells/mm^2^). **g** Confluent sheets of inflammatory cells (arrowheads) around the glands without tissue destruction or lymphoid nodule/follicle formation (100–500 cells/mm^2^). **h** Confluent sheets of inflammatory cells with lymphoid nodule formation (arrows) lie within stroma around the glands (> 500 cells/mm^2^). **i.** No signs of inflammation. **j** Urinary bladder surface epithelium is partially diminished (arrow) and few inflammatory cells (arrowhead) are seen under urothelium in the lamina propria (< 100 cells/mm^2^). **k** Urinary bladder surface epithelium is totally diminished (arrows), and there is lymphoid cell infiltration in the lamina propria and through the bladder wall (arrowheads) without tissue destruction or lymphoid nodule/follicle formation (100–500 cells/mm^2^). The scale bar in each image indicates 200 µm

After the cystometry experiments, the dorsal and ventral prostate lobes as well as the urinary bladder were excised and the rats were euthanized. Total prostate weight was noted and paraformaldehyde (4% in 0.1 M phosphate-buffer solution) was used to fix the tissues before histopathological analysis. After fixation (48–72 h), the tissues were embedded in paraffin and sectioned into 8 µm thin tissue sections (Histolab Products AB, Gothenburg, Sweden). Haematoxylin–eosin staining was performed and the histopathological analysis followed the same procedure as previous studies [13, 21], including the use of a similar grading scale (see Table 1). Prostatic inflammation was scored based on the magnitude or density of lymphocytes (grade) and the extent or distribution of lymphocyte infiltration (extent). The grade of inflammation was scored from 0 to 3 where 0 represented no inflammation and 3 represented severe inflammation while the extent of inflammation was scored as focal (1), multifocal (2) or diffuse (3), respectively (Fig. 2).

**Table 1:** Immunohistochemical grading scales

*Grade (Inflammatory cell density, cells/mm*^2^)	
0	No inflammation
1, mild	Individual inflammatory cells (< 100 cells/mm^2^)
2. moderate	Confluent sheets of inflammatory cells without tissue destruction or lymphoid follicle formation (100–500 cells/mm^2^)
3, severe	Confluent sheets of inflammatory cells with tissue destruction or lymphoid follicle formation (> 500 cells/mm^2^)
*Extent (Tissue area involved by inflammatory cell infiltrates)*	
1, focal	< 10%
2, multifocal	10–50%
3, diffuse	> 50%

Grading scales used to score inflammatory changes in prostate and urinary bladder. The procedure for determination of grade and extent of inflammation was adapted from Inamura et al. [21]

### Statistics

Statistical measurements were performed using GraphPad Prism version 8.2.1 (GraphPad Software Inc., San Diego, USA). One-way ANOVA or two-way ANOVA followed by Tukey`s correction for multiple comparisons was used for statistical comparisons of metabolic cage and cystometry findings, as these data were assumed to be normally distributed. Histopathological findings were statistically compared using the non-parametric Kruskal–Wallis test followed by Dunn`s test for multiple comparisons, to compare the mean ranks between each of the treatment groups and controls. Statistical significance was regarded for *p*-values < 0.05. Metabolic cage and cystometry data are presented as mean ± SEM while histopathological data are presented as median with range.

## Results

### Metabolic cage

There were no significant differences between the groups regarding total water intake (*p* = 0.299) or total amount of voided urine (*p* = 0.735) during the metabolic cage experiments (Table 2). The number of micturitions per hour were significantly higher in the L14 group as compared to controls (*p* = 0.035). Similarly, the total number of micturitions (over 16 h) were higher in the L14 group as compared to controls (*p* = 0.035, Table 2). No significant differences were seen between the L10 and L14 groups regarding the number of micturitions per hour or the total number of micturitions. The volume/micturition was significantly lower in both L10 and L14 as compared to controls (L10 vs control, *p* = 0.016; L14 vs control, *p* = 0.018). Again, there was no significant difference between the L10 and L14 group (Table 2).

**Table 2:** Metabolic cage results

	Control	L10	L14
Total water intake (mL)	7.50 ± 1.12	9.83 ± 2.40	11.83 ± 1.94
Total urine output (mL)	11.92 ± 1.34	11.67 ± 1.09	13.50 ± 2.28
Total number of micturitions	6.50 ± 0.43	13.50 ± 1.41	16.50 ± 4.15*
Micturitions/hour	0.41 ± 0.03	0.84 ± 0.09	1.03 ± 0.26*
Volume/micturition (mL)	1.62 ± 0.23	0.90 ± 0.10*	0.91 ± 0.13*

Comparisons between data from saline-treated controls and 10 (L10) and 14 (L14) days after intraprostatic injection with lipopolysaccharide (LPS)

*Denotes significant difference (*p* < 0.05) between control and treatment group; n = 6 per group. No significant differences were noted between the L10 and L14 groups

### Cystometry

**Fig. 3:**
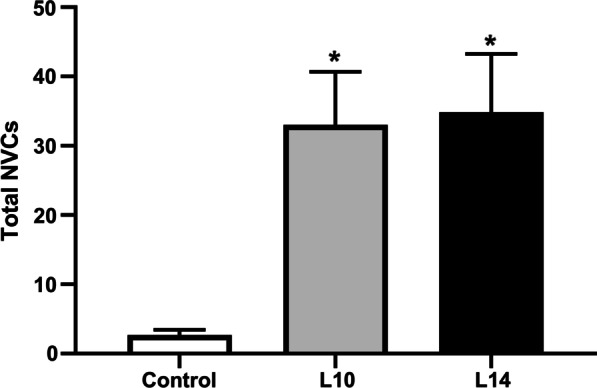
Total number of non-voiding contractions (NVCs) during cystometry. The observed number of NVCs were low in saline-treated controls. Following intraprostatic injection with LPS, the number of NVCs were significantly increased after 10 (L10) and 14 (L14) days. * denotes significant difference (*p* < 0.05) between control and treatment group; n = 6 per group

**Fig. 4:**
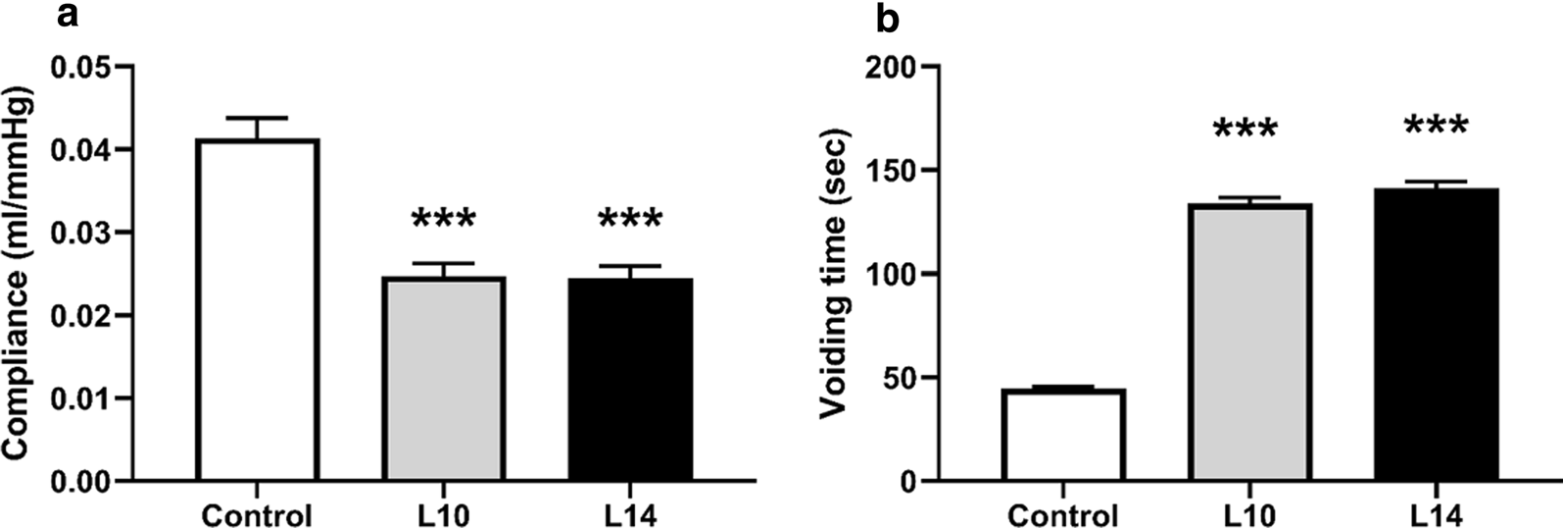
Bladder compliance (∆V/∆P) and voiding times during cystometry. To calculate compliance, the bladder was infused with saline while continuously monitoring intravesical pressure. **a** Bladder compliance was significantly decreased at 10 (L10) and at 14 (L14) days after intraprostatic injection with LPS. **b** Voiding times were significantly increased 10 (L10) and 14 (L14) days after intraprostatic injection with LPS. *** denotes significant difference (*p* < 0.001) between control and treatment groups; n = 6 per group

Baseline urinary bladder pressures were similar in all groups, both before and after the administration of agonist (MeCh and ATP). However, there were significantly more NVCs in the CP/CPPS groups as compared to controls (Fig. 3; control vs L10: 2.67 ± 0.76 vs 33.00 ± 7.67, *p* = 0.014; control vs L14: 2.67 ± 0.76 vs 34.83 ± 8.43, *p* = 0.009). No significant difference was seen in regard to NVCs between the CP/CPPS groups. Similarly, lower bladder compliance (∆V/∆P) was observed in the CP/CPPS groups as compared to controls (control vs L10: 0.041 ± 0.002 vs 0.025 ± 0.001, *p* < 0.0001; control vs L14: 0.041 ± 0.002 vs 0.024 ± 0.001, *p* < 0.0001; Fig. 4a). Rats in the CP/CPPS groups had significantly longer voiding times compared to controls (L10 vs control, 133.70 s ± 2.99 vs 44.61 s ± 0.81, *p* < 0.0001; L14 vs control, 141.2 s ± 3.19 vs 44.61 s ± 0.81, *p* < 0.0001; Fig. 4b). There were no significant differences between the CP/CPPS groups in regard to voiding time or compliance.

**Fig. 5:**
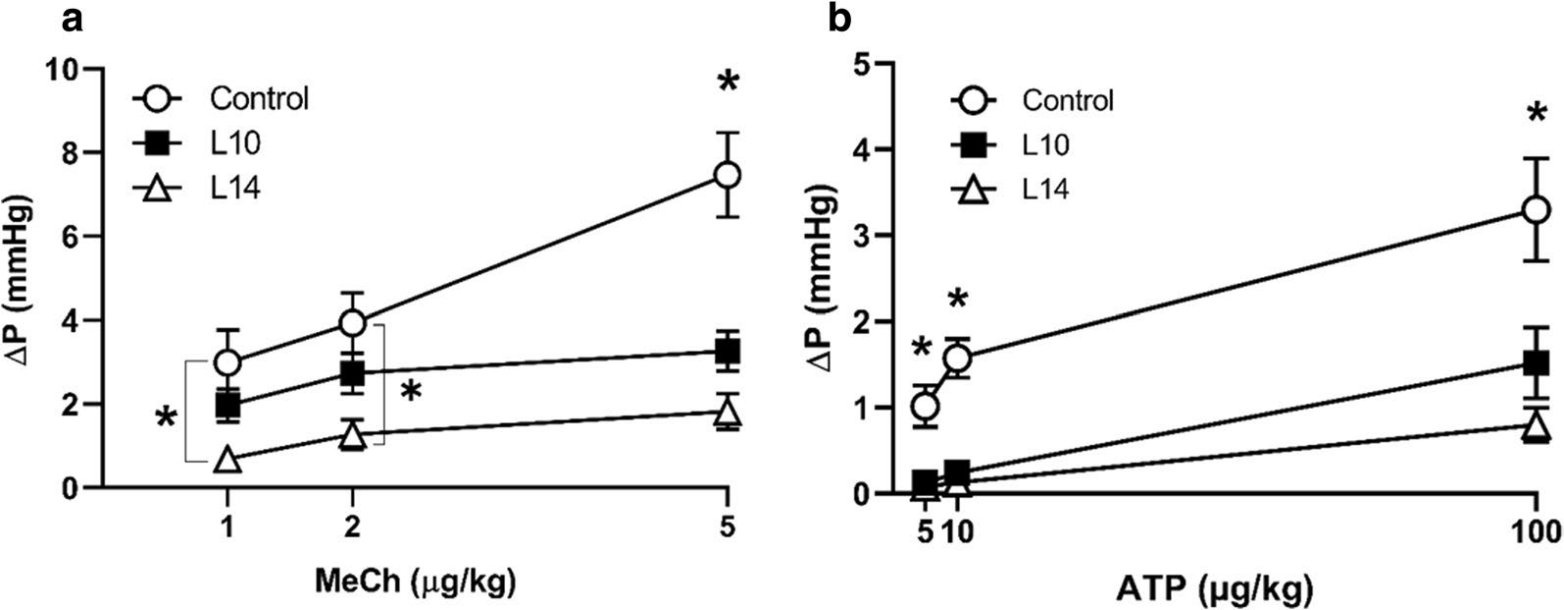
Intravesical pressure changes (∆P) upon agonist stimulation. Bladder contraction as a response to **a** methacholine (MeCh;1, 2 and 5 µg * kg^−1^ i.v.) and **b** ATP (5, 10 and 100 µg * kg^−1^ i.v.) in saline-treated controls (○) and 10 (L10; ■) and 14 (L14; ∆) days post intraprostatic injection with LPS. * denotes significant difference (*p* < 0.05) between control and treatment groups; n = 6 per group

Significantly decreased contractile responses (intravesical pressure change, ∆P) to MeCh were seen in the L14 group as compared to controls (at 1 µg * kg^−1^: L14 vs control, 0.69 ± 0.14 vs 2.98 ± 0.79, *p* = 0.013; at 2 µg * kg^−1^: L14 vs control, 1.27 ± 0.35 vs 3.93 ± 0.72, *p* = 0.004). However, there were no significant differences between the L10 group and controls in regards to ∆P at the same doses. At a dose of 5 µg * kg^−1^ MeCh, a significantly decreased response was seen in both the L10 and L14 groups, as compared to controls (Fig. 5a; L10 vs control, 3.26 ± 0.48 vs 7.47 ± 1.01, *p* < 0.0001; L14 vs control, 1.82 ± 0.43 vs 7.47 ± 1.01, *p* < 0.0001).

The contractile responses to all doses of ATP (5, 10 and 100 µg * kg^−1^) were significantly decreased in both the L10 and L14 groups as compared to controls (Fig. 5b; at 5 µg * kg^−1^: L10 vs control, 0.12 ± 0.04 vs 1.01 ± 0.24, *p* = 0.002; L14 vs control, 0.08 ± 0.03 vs 1.01 ± 0.24, *p* = 0.001; at 10 µg * kg^−1^: L10 vs control, 0.24 ± 0.09 vs 1.58 ± 0.23, *p* < 0.0001; L14 vs control, 0.13 ± 0.05 vs 1.58 ± 0.23, *p* < 0.0001; at 100 µg * kg^−1^: L10 vs control, 1.52 ± 0.41 vs 3.30 ± 0.60, *p* < 0.0001; L14 vs control, 0.80 ± 0.20 vs 3.30 ± 0.60, *p* < 0.0001).

### Histopathology

**Fig. 6:**
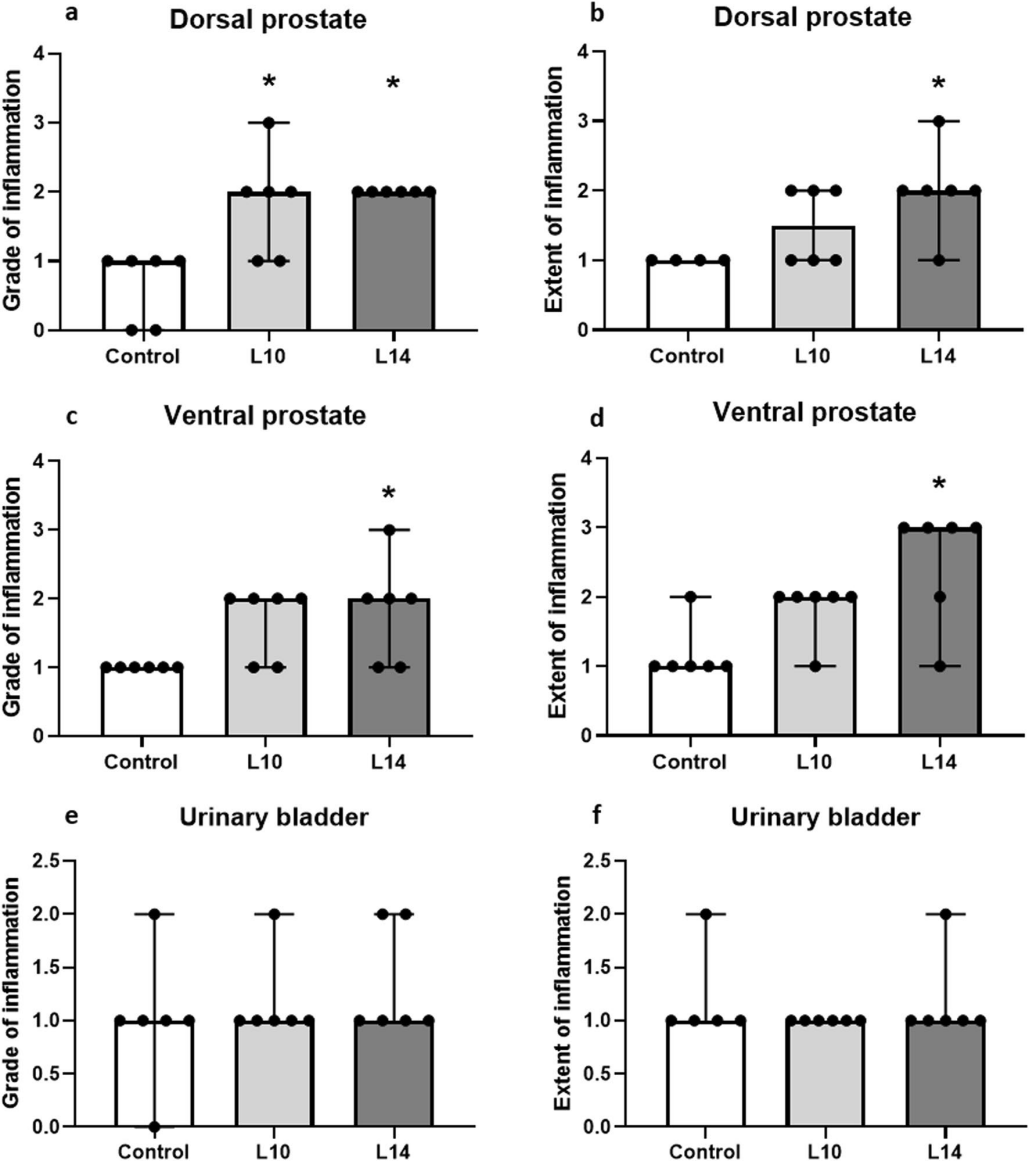
Histopathological analysis of prostate and bladder inflammation. Scatter plots with bars indicate the grade of inflammation (**a**, **c**, **e**) and the extent of inflammation (**b**, **d**, **f**) in discrete individual values in controls and 10 (L10) or 14 (L14) days after intraprostatic injection with LPS. Analysis was performed on tissue slices from dorsal prostate (**a-b**), ventral prostate (**c–d**) and urinary bladder (**e–f**). Extent of inflammation was graded for all tissues that display a grade of inflammation > 0. * denotes significant difference (*p* < 0.05) between the treatment groups and controls in terms of mean rank values (Dunn`s test); n = 6 per group

Prostate weight was not significantly altered in the L10 and L14 groups, as compared to controls (L10 vs control, 0.864 ± 0.037 vs 0.758 ± 0.038, *p* = 0.113; L14 vs control, 0.888 ± 0.042 vs 0.758 ± 0.038, *p* = 0.063). Grade of inflammation in the dorsal prostate was significantly higher in the L10 and L14 groups as compared to controls (Fig. 6a). The extent of inflammation in the dorsal prostate was higher in the L14 group as compared to controls (Fig. 6b). Both grade (Fig. 6c) and extent (Fig. 6d) of prostatic inflammation in the ventral prostate were significantly higher in the L14 group as compared to controls. Regarding the bladder, the grade (Fig. 6e) and extent (Fig. 6f) of inflammation were similar between all groups.

## Discussion

In the present in vivo study it was investigated if a primary intraprostatic infectious focus could be an inducing factor for an inflammatory state mimicking CP/CPPS. Further, the potential effects of CP/CPPS were examined on both afferent (NVCs, compliance) and efferent (voiding time, cholinergic and purinergic contractile responses) nervous control of bladder function as well as on possible inflammatory changes in the bladder. Metabolic cage experiments confirmed experimentally induced bladder overactivity and cystometry investigations showed significant negative effects of CP/CPPS on both afferent and efferent signalling, without any demonstration of significant inflammatory changes in the bladder. Taken together, these data show that an initial infectious focus in the prostate can provoke prostate-to-bladder cross-organ sensory pathways in the pelvic region. These observations may be of clinical relevance as cross-organ sensitization in the pelvic region is believed to be a contributing cause for the frequently observed unsatisfactory treatment of men with CP/CPPS. Previous studies advocated that CP/CPPS could be associated with bacterial colonization in the prostate, at least in some patients [1, 4]. Rudick et al. speculated that characteristics of the pathogen and immunogenetic background of the host could be important key features in determining the development of chronic pelvic pain after an initial infectious focus in the prostate [1]. The outcomes of the present study support the idea that a primary infectious focus in the prostate could be a more imperative cause of CP/CPPS than previously assumed.

The current findings confirm that induction of CP/CPPS by intraprostatic injection with LPS was a valid choice of method. Metabolic cage experiments showed increased urinary frequency in rats with LPS-induced CP/CPPS, which could be interpreted as CP/CPPS having a sensitizing effect on bladder afferent signalling. This finding was in concordance with the outcomes of a previous study [13]. Likewise, cystometry data revealed an increased number of NVCs in CP/CPPS groups, as well as decreased compliance, which is in line with the findings in previous studies that investigated the effect of chemically induced prostate inflammation on bladder function [10, 13]. Although no significant differences between the groups regarding prostate weight could be observed, histopathological examination of the prostate tissues revealed chronic inflammation following LPS injection.

Voiding times were significantly longer in both LPS-injected groups, as compared to controls. This is interpreted as a demonstration of the effect of CP/CPPS on efferent signalling. However, the increase in voiding times in CP/CPPS groups could also be due to bladder outlet obstruction, potentially due to an increase in innate urethral contractile activity or BPH. Some previous studies have shown a correlation between chronic prostate inflammation and BPH [2]. Chronic inflammation in the prostate is also related with a higher risk for disease progression and acute urinary retention in men with BPH [2, 22]. However, as previously mentioned, no significant differences were seen between the groups regarding prostate weight.

Previous studies have shown reduced muscarinic receptor-induced bladder contractility following induction of hemorrhagic cystitis [23, 24]. This is in line with the findings in the present study. Further, the current ATP-evoked purinergic bladder contractions were reduced in both CP/CPPS groups, which is consistent with the findings in a recent study on chemically induced prostatitis [13]. However, reduced purinergic responses are not in line with previous studies that investigated bladder activity during cystitis [25–27]. This discrepancy could be explained by the simple fact that the current study is not a cystitis model. On the contrary, the current histopathological data highlight the absence of significant changes between the groups regarding bladder inflammation. Another contributing factor could be alterations in the cholinergic component of ATP-evoked bladder contractility [24]. The reduced muscarinic and purinergic bladder contractile responses that are observed in the present study, despite likely simultaneous afferent sensitization, indicate that prostate-to-bladder cross-sensitization can lead to local changes in the detrusor, urothelium and/or on efferent nerves.

The histopathological analysis showed higher scores for both the grade and extent of inflammation in the ventral as well as the dorsal prostate following LPS injection. This finding is in line with a recent study in which zymosan was used to create a model of chemically induced CP/CPPS [13]. However, dissimilar from the previous study, no increase in bladder inflammation scores could currently be observed. Much of the recent literature is controversial regarding the existence of bladder inflammation after induced prostatitis in animal models [6, 10, 13]. This dissimilarity between different studies is likely due to the different substances being used to induce prostatitis and the variety of inflammation evaluation methods. Regarding this, it is important to point out that the present study has some potential limitations. First, no pain evaluation was performed. Such an evaluation could have been beneficial to support the conclusion of induced CP/CPPS. Second, cystometry was performed under deep anaesthesia with isoflurane, which likely influenced nerve transmission. However, cystometry, including induction and maintenance of anaesthesia, was performed similarly in all groups, thus allowing valid comparisons between the groups. Third, there is a small risk that the surgical procedure that is required for the intraprostatic injection could cause minor injury to the pelvic floor, possibly affecting the bladder. Nevertheless, the same surgical procedure was performed in all groups. In addition, the prostate and bladder are in close proximity anatomically. However, the histopathological analysis did not reveal any significant inflammatory changes in the bladder which indicates that this potential pitfall was avoided.

In order to manage patients with inflammatory pain syndromes adequately it is crucial to understand the potential mechanisms underlying cross-sensitization between adjacent organs in the pelvic and abdomen region. Grundy et al. showed in a mouse model that colitis induced neuroplasticity in sensory pathways innervating the colon and bladder, resulting in bladder dysfunction [28]. It was further shown that the changes in bladder function were not due to bladder inflammation. Similar to this, the present study demonstrates induced changes in bladder function that do not correlate with inflammatory changes in the bladder. Instead, alterations in both afferent and efferent signalling are indicated. Despite not performing retrograde labelling or measuring nerve activity per se, this sheds a light on the possible mechanisms of cross-sensitization between the prostate and urinary bladder. However, the exact underlying mechanisms of prostate-to-bladder cross-organ sensitization are yet to be fully elucidated and require further investigation.

## Conclusions

The model of LPS-induced chronic prostatitis used in the current study was shown to be valid for examining prostate-to-bladder cross-organ sensitization. It was shown that prostate-to-bladder sensitization could be triggered by an initial bacterial focus in the prostate, which upon development of chronic prostatitis gave rise to overactive bladder and alterations in both afferent and efferent signalling. Further clinical and intervention studies are needed to elucidate the underlying mechanisms of prostate-to-bladder cross-organ sensitization and improve the unsatisfactory management of men with CP/CPPS.

## Data Availability

All original data included in this study will be made available from the corresponding author upon reasonable request.

## Abbreviations

ATP: Adenosine-5′-triphosphate
CP/CPPS: Chronic prostatitis/chronic pelvic pain syndrome
IC/BPS: Interstitial cystitis/bladder pain syndrome
IL-1: Interleukin-1
IL-6: Interleukin-6
LPS: Lipopolysaccharide
LUTS: Lower urinary tract symptoms
MeCh: Methacholine
NVCs: Non-voiding contractions
TNF-α: Tumor necrosis factor alpha

## References

[1] Rudick CN, Berry RE, Johnson JR, Johnston B, Klumpp DJ, Schaeffer AJ (2011). Uropathogenic *Escherichia coli* induces chronic pelvic pain. Infect Immun.

[2] Dos Santos Gomes FO, Oliveira AC, Ribeiro EL, da Silva BS, Dos Santos LAM, de Lima IT (2018). Intraurethral injection with LPS: an effective experimental model of prostatic inflammation. Inflamm Res.

[3] Leskinen MJ, Rantakokko-Jalava K, Manninen R, Leppilahti M, Marttila T, Kylmälä T (2003). Negative bacterial polymerase chain reaction (PCR) findings in prostate tissue from patients with symptoms of chronic pelvic pain syndrome (CPPS) and localized prostate cancer. Prostate.

[4] Nickel JC, Alexander RB, Schaeffer AJ, Landis JR, Knauss JS, Propert KJ (2003). Leukocytes and bacteria in men with chronic prostatitis/chronic pelvic pain syndrome compared to asymptomatic controls. J Urol.

[5] Funahashi Y, Wang Z, O’Malley KJ, Tyagi P, DeFranco DB, Gingrich JR (2015). Influence of *E. coli*-induced prostatic inflammation on expression of androgen-responsive genes and transforming growth factor beta 1 cascade genes in rats. Prostate.

[6] Funahashi Y, Takahashi R, Mizoguchi S, Suzuki T, Takaoka E, Ni J (2019). Bladder overactivity and afferent hyperexcitability induced by prostate-to-bladder cross-sensitization in rats with prostatic inflammation. J Physiol.

[7] Kim HJ, Park JW, Cho YS, Cho CH, Kim JS, Shin HW (2013). Pathogenic role of HIF-1alpha in prostate hyperplasia in the presence of chronic inflammation. Biochim Biophys Acta.

[8] Wong L, Hutson PR, Bushman W (2014). Prostatic inflammation induces fibrosis in a mouse model of chronic bacterial infection. PLoS ONE.

[9] Song B, Jiang C, Wang Y, Lu Y, Li L (2009). Newly found prostate-bladder neural reflex in rats–possible mechanism for voiding dysfunction associated with prostatitis/pelvic pain. Urology.

[10] Schwartz ES, La JH, Young EE, Feng B, Joyce S, Gebhart GF (2016). Chronic prostatitis induces bladder hypersensitivity and sensitizes bladder afferents in the mouse. J Urol.

[11] Lee S, Yang G, Xiang W, Bushman W (2016). Retrograde double-labeling demonstrates convergent afferent innervation of the prostate and bladder. Prostate.

[12] Chen Y, Wu X, Liu J, Tang W, Zhao T, Zhang J (2010). Distribution of convergent afferents innervating bladder and prostate at dorsal root Ganglia in rats. Urology.

[13] Aydogdu O, Gocun PU, Aronsson P, Carlsson T, Winder M (2021). Prostate-to-bladder cross-sensitization in a model of zymosan-induced chronic pelvic pain syndrome in rats. Prostate.

[14] Patnaik SS, Lagana AS, Vitale SG, Buttice S, Noventa M, Gizzo S (2017). Etiology, pathophysiology and biomarkers of interstitial cystitis/painful bladder syndrome. Arch Gynecol Obstet.

[15] Arora HC, Shoskes DA (2015). The enigma of men with interstitial cystitis/bladder pain syndrome. Transl Androl Urol.

[16] Forrest JB, Nickel JC, Moldwin RM (2007). Chronic prostatitis/chronic pelvic pain syndrome and male interstitial cystitis: enigmas and opportunities. Urology.

[17] Zhang J, Liang C, Shang X, Li H (2020). Chronic prostatitis/chronic pelvic pain syndrome: a disease or symptom? Current perspectives on diagnosis, treatment, and prognosis. Am J Mens Health.

[18] Andersson M, Aronsson P, Giglio D, Wilhelmson A, Jerabek P, Tobin G (2011). Pharmacological modulation of the micturition pattern in normal and cyclophosphamide pre-treated conscious rats. Auton Neurosci.

[19] Patel B, Perez F, Aronsson P, Alothmani R, Carlsson T, Winder M (2020). Combination drug therapy against OAB normalizes micturition parameters and increases the release of nitric oxide during chemically induced cystitis. Pharmacol Res Perspect.

[20] Andersson MC, Tobin G, Giglio D (2008). Cholinergic nitric oxide release from the urinary bladder mucosa in cyclophosphamide-induced cystitis of the anaesthetized rat. Br J Pharmacol.

[21] Inamura S, Ito H, Shinagawa T, Tsutsumiuchi M, Taga M, Kobayashi M (2018). Prostatic stromal inflammation is associated with bladder outlet obstruction in patients with benign prostatic hyperplasia. Prostate.

[22] Sciarra A, Di Silverio F, Salciccia S, Autran Gomez AM, Gentilucci A, Gentile V (2007). Inflammation and chronic prostatic diseases: evidence for a link?. Eur Urol.

[23] Barut EN, Engin S, Barut B, Kaya C, Kerimoglu G, Ozel A (2019). Uroprotective effect of ambroxol in cyclophosphamide-induced cystitis in mice. Int Urol Nephrol.

[24] Stenqvist J, Winder M, Carlsson T, Aronsson P, Tobin G (2017). Urothelial acetylcholine involvement in ATP-induced contractile responses of the rat urinary bladder. Eur J Pharmacol.

[25] Burnstock G (2014). Purinergic signalling in the urinary tract in health and disease. Purinergic Signal.

[26] Kumar V, Chapple CR, Surprenant AM, Chess-Williams R (2007). Enhanced adenosine triphosphate release from the urothelium of patients with painful bladder syndrome: a possible pathophysiological explanation. J Urol.

[27] Sun Y, Chai TC (2006). Augmented extracellular ATP signaling in bladder urothelial cells from patients with interstitial cystitis. Am J Physiol Cell Physiol.

[28] Grundy L, Harrington AM, Castro J, Garcia-Caraballo S, Deiteren A, Maddern J, et al. Chronic linaclotide treatment reduces colitis-induced neuroplasticity and reverses persistent bladder dysfunction. JCI Insight. 2018;3(19). 10.1172/jci.insight.121841 PMC6237488 30282832

